# Combining ability for quantitative traits related to productivity in durum wheat

**DOI:** 10.18699/VJGB-22-63

**Published:** 2022-10

**Authors:** R.G. Dragov

**Affiliations:** Agricultural Academy, Field Crops Institute, Chirpan, Bulgaria

**Keywords:** gene action, combining ability, quantitative traits, durum wheat, diallel cross, действие генов, комбинационная способность, количественные признаки, твердая пшеница, диаллельное скрещивание

## Abstract

The present study was to determine the nature of gene action and combining ability of six quantitative traits related to productivity of five varieties and ten hybrid combinations of durum wheat. Five modern durum wheat varieties were used in diallel crosses as parents. The study includes three F1 and two F2 generations. The experiments were done in a randomized block design in three replications during three years. Significant differences between the genotypes in both generations was found for all the traits. The general combining ability and specific combining ability showed reliability in both generations. Obtained results suggests that breeding schemes should include both types of genetic effects in order to improve productivity components. The ratio of variances showed that general combining ability has a greater influence on the inheritance of plant height, spike length and thousand kernels weight. For productivity tillering capacity, number of spikelets per spike and kernels weight per spike, specific combining ability has a great impact in inheritance. For thousand kernels weight a redetermination of the genetic formula was established in both generations. Durum wheat varieties Deni, Superdur and Progres were found to be the best general combinators for studied productivity elements. The most valuable cross combinations were Deni × Superdur, Superdur × Predel and Progres × Predel. Parental wheat varieties and progenies from these crosses can be used for improving productivity components and for increasing yields in durum wheat breeding programs.

## Abstract (ru)

Настоящее исследование направлено на определение характера действия генов и комбинационной способности твердой пшеницы по шести количественным признакам для пяти сортов и десяти гибридных комбинаций. В исследование были включены пять современных сортов твердой пшеницы в качестве родителей при диаллельном скрещивании. Изучены три поколения F1 и два поколения F2. Эксперименты выполнены в рандомизированном блочном дизайне в трех повторeниях в течение трех лет. Выявлены достоверные различия между генотипами по всем изучаемым признакам в обоих поколениях. Общая комбинационная способность и специфическая комбинационная способность показали достоверные отличия в обоих поколениях. Полученные результаты позволяют предположить, что селекционная схема должна учитывать оба типа генетических эффектов для улучшения элементов продуктивности. Соотношение дисперсий демонстрирует, что общая комбинационная способность больше влияет на наследуемость признаков «высота растения», «длина колоса» и «масса 1000 зерен». Для признаков «продуктивная кустистость», «число колосков в колосе» и «масса зерен в колосе» большее влияние на наследование оказывает специфическая комбинационная способность. Для массы 1000 зерен в обоих поколениях установлено переопределение генетических формул. Лучшими общими комбинаторами по элементам продуктивности одновременно по нескольким признакам являются сорта Дени, Супердур и Прогрес. Самые ценные комбинации скрещиваний по нескольким признакам – Дени × Супердур, Супердур × Предел и Прогрес × Предел. Эти сорта и комбинации можно использовать для улучшения признаков продуктивности и повышения урожайности в программах селекции твердой пшеницы.

## Introduction

Breeding strategy of durum wheat is based on genetic information on the inheritance of the main quantitative traits related to productivity. To obtain such information, it is necessary to apply a genetic model corresponding to the source material to be used. In a regular breeding program, it is important to identify the best parents for hybridization and crosses to select valuable genotypes (Inamullah et al., 2006). Diallel crosses have been used for a long time in genetic research to determine the inheritance of a trait among a set of genotypes and to identify superior parents for hybrid or varieties development.

Information on additive gene effects i. e. general combining ability effects (GCA) is of great importance, because it successfully predicts the genetic potential of parents who give desired results in segregating generations. In determining the specific combining ability effects (SCA), a relationship is established with the non-additive gene effects (dominance and epistasis components). The identification of a good hybrid combination with high SCA on a given trait makes it possible to expect a more probable transgressive form for the trait. Combining ability describes the breeding value of parental varieties to produce better hybrids as well as their crosses (Griffing, 1956).

The importance of combining ability is related to the evaluation of parental lines and their hybrids by their respective additive and non-additive genetic effects in relation to a certain trait. Diallel crosses give a more general view of combining ability, where general and specific combining ability are indicators for nature of gene action (Farooq et al., 2010). Assesment of GCA effects show that it is not possible to choose a good general combiner for all traits of the productivity. This is due to the inability to combine in one genotype high GCA on all traits (Kashif et al., 2008). However, some parents show desired GCA effects for several traits. It is obvious that highyielding varieties included in crosses are mainly responsible for increasing productivity (Adel, Ali, 2013).

A number of authors, using schemes of full and half diallel crosses, have established the breeding value of a large number of varieties and the gene action for traits related to productivity. In the publications cited below, the authors found that both additive and non-additive gene effects played a role in the inheritance of tested traits. According to J. Yao et al. (2011) and M. Singht et al. (2018) plant height and spike length mainly controlled by additive gene effects. It was reported that in the inheritance of thousand kernels weight the non-additive gene effects play an essential role (Akinci, 2009; Pansuriya et al., 2014), while A. Hannachi et al. (2017) and A. Hassan et al. (2018) establish inverse. Plant height, tillering capacity and number of spikelets per spike were mainly controlled by non-additive gene effects (Adel, Ali, 2013; Pansuriya et al., 2014; Kandil et al., 2016), when A. Hannachi et al. (2017) reported that plant height and productivity tillering capacity were additive. The inheritance of the spike length, number of spikelets per spike, and kernel weight per spike are controlled by non-additive gene effects and they have a major role (Patel et al., 2016; Tiwari et al., 2017), also A. Pansuriya et al. (2014) for these traits and for spike length. Productivity tillering capacity and number of spikelets per spike are controled by non-additive gene effects, on the other hand, for spike length and thousand kernel weight additive gene effects dominate in inheritance (Farooq et al., 2019).

It can be concluded from the published data that parental varieties have a great influence on both types of combining ability. On the other hand, they are carried out in different growing conditions, which gives additional confirms to this statement of diverstity

The present investigation was undertaken to determine the nature and magnitude of gene action and general and specific combining ability for five modern durum wheat varieties and for six quantitative traits related to productivity in diallel cross of durum wheat

## Materials and methods

Parents and crosses. Five modern durum wheat varieties were included in the study as the parental varieties in the half diallel crosses. The varieties are selected among the new Bulgarian varieties of durum wheats, including the old and the new variety-standard and the Austrian variety Superdur, which has recently become widespread in Bulgaria. Victoria – Bulgaria, Deni – Bulgaria, Superdur – Austria, old variety-standard Progres – Bulgaria and new variety-standard Predel – Bulgaria. The choice of varieties is based on their previous observation. They are created in Field Crops Institute, Chirpan and are genetically distant. Progres and Deni are created by experimental mutagenesis combined with hybridization and Victoria and Predel are created by hybridization. A diallel cross was performed in which all the described varieties were crossed with each other without reciprocal combinations. The crosses was carried out handmade at the beginning of heading time in field condition. The following ten combinations were performed: Victoria × Deni, Victoria × Superdur, Victoria × Progres, Victoria × Predel, Deni × Superdur, Deni × Progres, Deni × Predel, Superdur × Progres, Superdur × Predel, Progres × Predel. From each combination, 30 spikes were castrated and pollinated. From the harvested F1 plants, the seeds necessary for sowing of F2 generation were randomly selected.

**Fig. 1.**
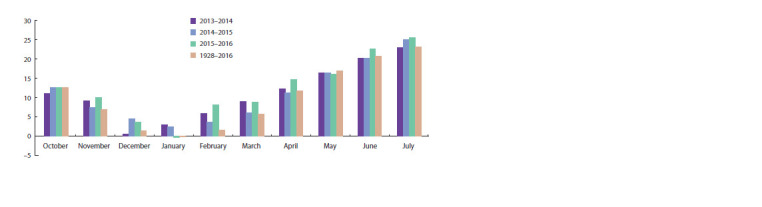
Average monthly and multiyear air temperature during 2013–2016 harvest years.

**Fig. 2.**
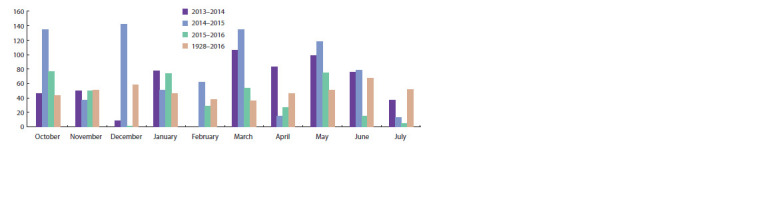
Average monthly and multiyear amount of precipitation during 2013–2016 harvest years.

Management. The parents are sown in each replication in two rows, the F1 hybrids in two rows, and the F2 hybrids in five rows. Genotypes are sown handmade in the field in beds. Row length – two meters, row spacing – twenty cm and inside the row – five cm in a randomized block design with three replications. After the full maturity phenophase, the necessary plants from each replication are harvested and collected for biometric research. Twenty plants were selected from the parents and F1 generation and thirty plants from F2 generation at random. The diallel cross was performed in three consecutive years. Thus, generation F1 for three years and F2 for two years are provided. The experiments was conducted in three harvest years 2014, 2015 and 2016. The experiments was carried out in the breeding field of the Field Crops Institute – Chirpan according to the adopted technology for growing durum wheat. The predecessor is spring peas. The soil type is Chernozems compact Eutric Vertisols (by FAO). The three years meteorological condition are characterized by higher temperatures compared to the multi-year period (Fig. 1). The first year have 18.5 % and the second 58.2 % precipitation over the multi-year period during the growing season, while in the third year precipitation are 17.5 % less than in the multi-year period (Fig. 2).

The following traits were observed. Plant height (cm) – it is measured from the ground surface to the end of the spike without the awns on the main stem in centimeters. Productivity tillering capacity (pcs.) – the fertile spikes of one plant are counted. Spike length (cm) – measured on the main stem from the base of the spike to the top of the uppermost spikelet. Number of spikelets per spike (pcs.) – the spikelets in the main spike are counted. Number of kernels per spike (pcs.) – all kernels of the main spike are counted after handmade threshing. Thousand kernels weight (g) – five hundred kernels are weighed and multiplied by two. All traits are determined by methodology by Y. Enchev et al. (1976).

Statistical analysis. The data from the three years F1 and the two years F2 are averaged and on them are conducted statistical processing. In the processing of the experimental data, mathematical and statistical methods were used on the results according to the set goal of the research. To perform diallel analysis was used combining ability analysis in diallel crosses – by method II model I (Griffing, 1956) with the program software of M. Burow and J. Coors (1994). Analysis of variance (ANOVA) by traits is derived through the same program on M. Burow and J. Coors (1994).

## Results

The results of analysis of variance showed statistically significant differences between the genotypes for all studied traits in both generations. The values of the variances for GCA and SCA were significant in both generations (Table 1). Therefore, both additive and non-additive gene effects (dominance and epistasis) were of significant importance in the inheritance of the traits. The studied traits related to durum wheat productivity show that they are controlled by both additive and non-additive gene effects.

**Table 1.**
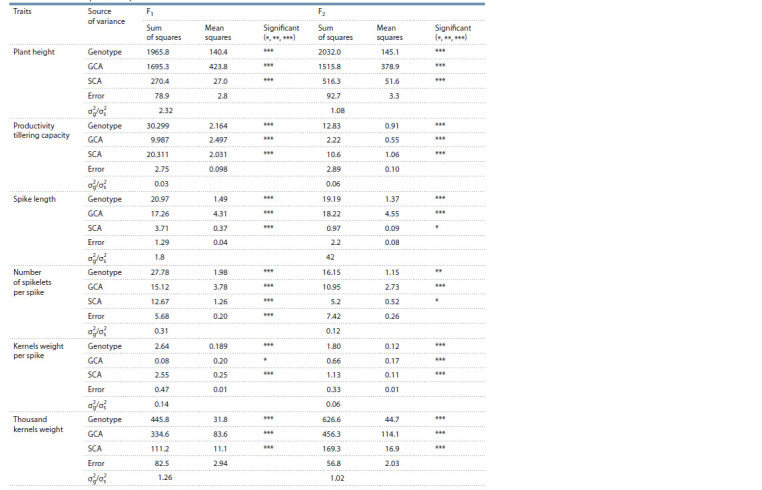
ANOVA for general combining ability (GCA), specific combining ability (SCA) and relation to variance of GCA and SCA (σ 2 g/σ 2 s ) for six traits related to productivity * p ≤ 0.05; ** p ≤ 0.01; *** p ≤ 0.001; σ 2 g – GCA variance; σ 2 s – SCA variance.

The ratio of GCA and SCA variances (σ 2 g/σ 2 s ) for F1 and F2 are presented in Table 1. For plant height, spike length, number of spikelets per spike and thousand kernel weight, the sum of squares indicates that additive gene effects have a greater influence in inheritance. For the other two traits, the sum of the squares indicates that non-additive gene effects have a greater impact. This is proved by the ratio of the variances of GCA and SCA, respectively. The preponderance of additive gene effects (σ 2 g/σ 2 s > 1) was found in the inheritance of plant height, spike length and thousand kernel weight. The spike length in F2 generation showed a significant increase, which indicates that the additivity increases. Domination of additive gene effects allow application of classical breeding methods. For these traits selection can start in early segregating generations (F2–F3).

Domination of non-additive gene effects (σ 2 g/σ 2 s < 1) is observed for the productivity tillering capacity, number of spikelets per spike and kernels weight per spike. Non-additive gene effects (dominance and epistasis) prevalence in their expression

This analysis does not allow to determine or dominance or epistasis are responsible for the inheritance of the traits. It is well known that when inheritance is determined by nonadditive genetic effects, selection in early segregating generations will be difficult. In this case effective selection must start in the later segregating generations F4–F5.

Although the preponderance of additive genetic effects for the thousand kernel weight in the individual years and generations has been established, there is a change in the genetic effects controlling the trait. This is due to the genotypeenvironment interaction and is explained by the phenomenon of redetermination of the genetic formula. In F1 in 2014 the non-additive genetic effects preponderance, and in 2015 and 2016 the additive ones. In F2 in 2015 the non-additive genetic effects preponderance and in 2016 the additive ones (data not shown). In the individual years in both generations, all other traits show a one-way ratio of variances that determine the influence of genetic effects.

The analysis for GCA of parents and SCA of hybrids for the studied traits in F1 and F2 is presented in the next two tables (Tables 2 and 3). From a breeding point of view, genotypes with a negative value for plant height due to the connection with lodging are more valuable. For all other traits, positive values are preferable, as their increase will lead to an increase in productivity.

**Table 2.**
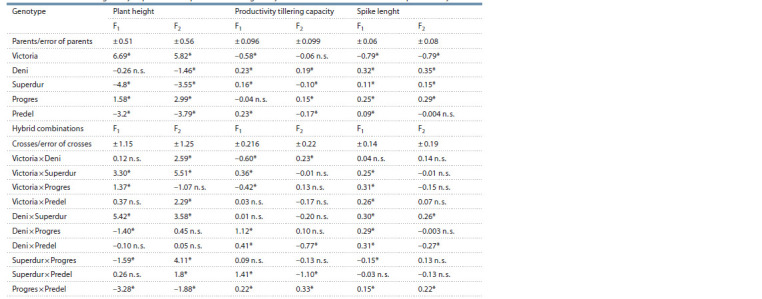
General combining ability of parents and specific combining ability of crosses for three traits related to productivity * p ≤ 0.05; n. s. – no significant.

**Table 3.**
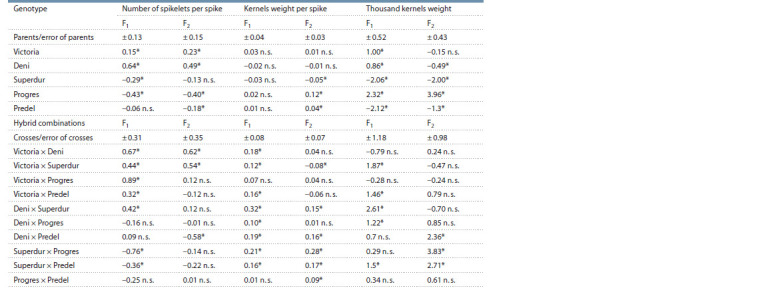
Values for general combining ability of parents and specific combining ability of crosses for three quantitative traits related to productivity * p ≤ 0.05; n. s. – no significant.

Plant height. Table 2 presents the values for plant height. The varieties Victoria and Progres has a significant and positive values for GCA in F1 and F2. They increase the plant height in the hybrids in which he participated as parents. The varieties Superdur and Predel have negative significant values of GCA in both generations. These varieties reducing the plant height in the hybrids in which they participate. They can be used successfully in the breeding program for obtaining dwarf durum wheats. In terms of SCA, valuable are the hybrid combination Progres × Predel, with significant negative values in both generations. The other crosses have different values for SCA, and in different generations they are differently significant and change their signs according to the generation.

Productivity tillering capacity. Table 2 presents the values for GCA and SCA for the productivity tillering capacity. Significant values of GCA to increase the trait of productivity tillering capacity have Deni variety in both generations. The varieties Superdur and Predel have positive and significant values in F1 generation, while in F2 generation the values are significant but negative. Variety Victoria has a significant and negative GCA in F1 and a negative and nonsignificant in F2 and reduces the values of the trait. Of greater interest are hybrid combinations and their SCA values (see Table 2), as non-additive effects have been found to preponderance. The results show that one of the crosses Progres × Predel has significant and positive values in both generations for SCA. The other crosses occupy an intermediate position.

Spike length. Table 2 presents the values for the general and specific combining ability of parents and hybrids for spike length. We define the varieties Deni, Superdur and Progres as good general combinators to increase spike length, as they have positive and significant values for GCA in both generations. Victoria variety has significant and negative values in both generations and it decreases the spike length in the hybrids in which it participates. The SCА values of the hybrids show that two crosses showed significant and positive values in F1 and F2 are Deni × Superdur and Progres × Predel

Number of spikelets per spike. The values for GCA and SCA for the trait number of spikelets per spike are presented in Table 3. The Victoria and Deni varieties in both generations have significant values to increasing number of spikelets per spike and they are good general combinators for this trait. Variety Progres has negative GCA and reducing the values of the trait. The other varieties have nonsignificant values, which shows their insignificant role. The greate interest is in hybrid combinations, as non-additive gene effects have been shown to play a major role in inheritance. The hybrid combinations Victoria × Deni and Victoria × Superdur show significant and positive SCA effects in both generations. With the highest SCA value is the cross Victoria × Deni.

Kernels weight per spike. Table 3 represents the values for parental GCA and hybrid SCA. In F1 there are no varieties with significant GCA effects. No good general combinators have been reported to increase kernels weight per spike in both generations. In the F2 generation, the Progres and Predel varieties increase the values of the kernels weight per spike, and the Superdur variety decreases it.

These results are very contradictory and it is difficult to define any of the varieties as a good general combiner on this trait. We can consider that the varieties Progres and Predel are good general combinators on the basis of showing significant and positive GCA effects in F2 generation. Greater attention should be paid to hybrid combinations, as SCA effects preponderance. Four significant good hybrid combinations are observed in terms of SCA effects. They are positive and significant in both generations. The most promising hybrid combinations are Deni × Superdur, Deni × Predel, Superdur × Progres and Superdur × Predel.

Thousand kernels weight. The variety Progres (see Table 3) is good general combiners for increasing the values of the trait thousand kernels weight. Variety Progres has positive and significant values in both generations. On the other hand, general combiners that have been significant to reduce grain size are the Predel and Superdur varieties in both generations. In Table 3 can be seen that the cross Superdur × Predel, which in both generations shows positive and significant values for the SCA effects, is interesting in terms of breeding. The remaining crosses in most cases have a significant value in only one of the generations. For all traits the values for GCA and SCA in most cases are in one-way direction and can be relied on their reliability

For the possibilities of heterosis in the breeding of durum wheat and obtaining transgressive forms, it is necessary to consider the crosses with significant SCA effects on several traits. Of the studied hybrid combinations (see Tables 2 and 3) as the most promising with significant SCA effects are Progres × Predel for plant height; Progres × Predel for productivity tillering capacity; Deni × Superdur and Progres × Predel for spike length; Victoria × Deni and Victoria × Superdur for the number of spikelets per spike; Deni × Superdur, Deni × Predel, Superdur × Progres and Superdur × Predel for grains weight per spike; Superdur × Predel for the thousand kernels weight.

## Discussion

Development of wheat varieties possessing improved yield related characters had been the major objective of durum wheat breeders. Thus availability of genetically based variation for traits like plant height, productivity tillering capacity, spike length, number of spikelets per spike, kernels weight per spike and thousand grain weight breeding population is essential. Present genetic material used here to generate information on genetic nature of these traits. A number of studies by other investigations are in line with the results obtained by us for GCA and SCA. Many researchers have also found significant GCA and SCA effects for the plant height (Topal et al., 2004; Pansuriya et al., 2014; Ali et al., 2018; Singh et al., 2018; Sharma et al., 2019; Ayoob, 2020); for productivity tillering ability (Topal et al., 2004; Akinci, 2009; Adel, Ali, 2013; Parveen et al., 2018; Talha et al., 2018; Bajaniya et al., 2019; Farooq et al., 2019; Hammam et al., 2020); for spike length (Topal et al., 2004; Yao et al., 2011; Pansuriya et al., 2014; Patel et al., 2016; Rajput, Kandalkar, 2018; Sadeghzadeh-Ahari et al., 2018; Khaled et al., 2020; Shamsabadi et al., 2020); for number of spikelets per spike (Adel, Ali, 2013; Pansuriya et al., 2014; Kandil et al., 2016; Patel et al., 2016; Saeed, Khalil, 2017; Parveen et al., 2018; Khaled et al., 2020); for kernels weight per spike (Topal et al., 2004; Adel, Ali, 2013; Mandal, Madhuri, 2016; Patel et al., 2016; Talha et al., 2018; Amin, Towfiq, 2019; Shamsabadi et al., 2020); for thousand kernels weight (Topal et al., 2004; Akinci, 2009; Desale, Mehta, 2013; Brahim, Mohamed, 2014; Motawea, 2017; Ali et al., 2018; Hassan et al., 2018; Ali, 2019; Khokhar et al., 2019; Sharma et al., 2019).

The obtained results for the GCA and SCA give a very clear idea of the control in the inheritance of the traits elements of the yield. The impact of additive and non-additive gene action in the inheritance of the structural elements of the yield shows that in order to maximize the productivity of durum wheat, a system should be used that includes both variances in simultaneously

Plant height and spike length are used for an individual selection by the classical methods. In both generations they are controlled by additive gene effects. In most cases thousand kernels weight is also controlled by additive genetic effects. This shows that it is possible for breeders to obtain better results in improving these traits. It should be noted that in the case of plant height, spike length and in most cases for thousand kernels weight, the selection may start in the earlier segregating generations F2–F3. Because they are controlled by additive genetic effects. It should be noted that the main structural elements of yield – spike length and in most cases thousand kernels weight are controlled by additive gene effects. Preponderance of additive gene effects in inheritance of plant height has been reported by a number of other researchers (Yao et al., 2011; Motawea, 2017; Ali et al., 2018; Hassan et al., 2018; Rajput, Kandalkar, 2018; Singh et al., 2018; Talha et al., 2018; Sharma et al., 2019; Ayoob, 2020); for spike length (Kandil et al., 2016; Motawea, 2017; Parveen et al., 2018; Rajput, Kandalkar, 2018; Sadeghzadeh-Ahari et al., 2018; Singh et al., 2018; Farooq et al., 2019; Sharma et al., 2019; Khaled et al., 2020; Shamsabadi et al., 2020); for thousand kernels weight (Hannachi et al., 2017; Motawea, 2017; Ali et al., 2018; Hassan et al., 2018; Ali, 2019; Amin, Towfiq, 2019; Farooq et al., 2019; Khokhar et al., 2019; Sharma et al., 2019).

For other three traits preponderance non-additive gene effect in this investigation was observed. Therefore, selection in early segregating generations will be difficult. In this case, it is recommended that an effective selection must start in the later segregating generations F4–F5 when the influence of the non-additive effects (dominance) decreases and the additivity increases. The results from this study for productivity tillering capacity are in line with those obtained by other authors (Desale, Mehta, 2013; Mostafa et al., 2014; Kandil et al., 2016; Ahmad et al., 2017; Saeed, Khalil, 2017; El-Gammaal, Morad, 2018; Parveen et al., 2018; Talha et al., 2018; Amin, Towfiq, 2019; Bajaniya et al., 2019; Farooq et al., 2019; Ayoob, 2020; Hammam et al., 2020); for number of spikelets per spike (Mostafa et al., 2014; Kandil et al., 2016; Ahmad et al., 2017; Saeed, Khalil, 2017; Tiwari et al., 2017; Parveen et al., 2018; Talha et al., 2018; Farooq et al., 2019; Ayoob, 2020; Khaled et al., 2020); for kernels weight per spike (Padhar et al., 2013; Mostafa et al., 2014; Kandil et al., 2016; Mandal, Madhuri, 2016; Tiwari et al., 2017; Talha et al., 2018; Amin, Towfiq, 2019, Shamsabadi et al., 2020).

For thousand kernel weight, a redermination of the genetic formula of the trait was found. This is due to the genotype-environment interaction. Redetermination of the genetic formula is especially evident in the case of quantitative traits that are controlled by a large number of small polygens significantly influenced by environmental conditions (Dragavtsev, Averyyanova, 1983). The presence of this phenomenon makes it difficult to lead an effective selection on the thousand kernel weight in different years and generations and the selection must be conducted longer (Dragavtsev, Averyyanova, 1983; Dragavtsev et al., 1984). When the phenomenon of redetermination of the genetic formula is observed in the individual years, different forms are selected, controlling the trait in the breeding process. This means that in different years valuable forms are selected in which the trait is controlled by both additive and non-additive genetic effects.

The deepening of the research allows to specify the methods of the applied breeding strategy and to optimize and increase the efficiency of the selection. The possibility of evaluating genotypes and their breeding value as a starting material for increasing productivity is also important. With the conducted research it is possible to get information about two of the most important moments in a successful breeding program – choosing parents for hybridization and leading a purposeful selection. The selection on a separate trait can increase the yield, but a more significant increase would be obtained by simultaneously comprehensively improving its elements.

Varieties that have significant GCA effects for more than one trait are of grait interest for breeding. The results for the respective traits are presented in Tables 2 and 3. Tables show which varieties are good combiners on the studied traits. Variety Victoria is a good general combiner on the trait number of spikelets per spike and a bad combiner for the traits plant height and spike length. The Deni variety is a good general combiner in terms of productivity tillering capacity, spike length and number of spikelets per spike. The Superdur variety is defined as a good combiner for plant height and spike length and a bad combiner for thousand kernels weight. Variety Progres shows significant and positive values for GCA for the traits spike length and thousand kernels weight, and is a bad combiner for number of spikelets per spike and plant height. Variety Predel is a good combiner for the trait plant height and a bad combiner for thousand kernels weight. A good general combiner at the same time on three traits is the Deni variety. Good general combinators on two traits at the same time are the varieties Superdur and Progres. Good general combinators on one trait are the varieties Victoria and Predel. Varieties Victoria and Progres are bad combiners on two traits. Varieties Superdur and Predel are bad combiners on one trait. The only exception is the Deni variety, which has no traits like a bad combiner. The varieties Deni, Superdur and Progres emerge as the best general combinators for the elements of the productivity on several traits at the same time. To increase the yield, it is necessary to simultaneously improve several valuable traits. The certain general combining abilities are a prerequisite for the correct selection of parental forms and their crossing for the purposes of the durum wheat breeding program.

As can be seen, the varieties bearing high GCA most often enter the crosses with high SCA. According to the various traits, there are good crosses, such as combined parents with high X high GCA and those who have combined parents with low X low GCA. Some with high SCA values are also a combinations of high X low GCA. As the most valuable hybrid combination with significant SCA effects on several traits its define Deni × Superdur, Superdur × Predel and Progres× Predel.

Determining the combining ability shows that it is not possible to have one variety can good combinator for all traits. Not all crosses with high SCA effects were obtained from the crosses of a good X good GCA parent (Kumar, Maloo, 2012). Rather, crosses with high SCA effects are obtained from crosses between bad X bad and bad X good combiner. They argue that such manifestations are due to the involvement of dominant or epistatis gene effects. Crosses with high SCA may be more likely to be sources of transgression (Gami et al., 2011; Tiwari et al., 2015). Transgressive lines on a certain traits can be a source for creating highly efficient durum wheat varieties. Evaluations of gene action explain the genetic potential of breeding materials and contribute to the targeted management of breeding progress in durum wheat productivity.

## Conclusion

The study found that both additive and non-additive gene effects are of significant importance in the nature of gene action of the productivity traits. This implies a breeding system that includes both gene effects for improving the elements of productivity. Inheritance of plant height, spike length and thousand kernels weight is mainly controlled by additive gene effects and it is possible to start selection of genotypes in the early segregating generations F2–F3. Inheritance of productivity tillering capacity, number of spikelets per spike and kernels weight per spike is controlled by non-additive gene effects. Therefore, the selection on these traits should start in the later segregating generations F4–F5. There is obtained a change in the genetic effects affecting the expression of the trait thousand kernels weight, which indicates the presence of the redetermination of the genetic formula.

## Conflict of interest

The authors declare no conflict of interest.
